# Association of Coronary Sinus Flow with Long-Term Risk of Acute Coronary Syndrome and Composite Cardiovascular Events

**DOI:** 10.3390/biomedicines14040808

**Published:** 2026-04-02

**Authors:** Ercan Akşit, Hasan Bozkurt, Mehmet Arslan

**Affiliations:** 1 Department of Cardiology, Faculty of Medicine, Çanakkale Onsekiz Mart University, Çanakkale TR-17100, Turkey; mehmet_arslan@outlook.com.tr; 2 Department of Cardiology, Ministry of Health, Gelibolu Şehit Koray Onay State Hospital, Çanakkale TR-17500, Turkey; drhasanbozkurt@gmail.com

**Keywords:** acute coronary syndrome, coronary sinus, myocardial infarction

## Abstract

**Background/Objectives:** Acute coronary syndrome (ACS) remains a leading cause of sudden cardiac death worldwide; however, limited data exist regarding the relationship between coronary arterial and coronary venous dynamics in this context. The present study aimed to evaluate whether coronary sinus flow (CSF) dynamics obtained via transthoracic echocardiography (TTE) are associated with the long-term risk of ACS and cardiovascular events. **Methods:** This retrospective observational cohort study included 100 patients who underwent elective coronary angiography and had their CSF parameters assessed via TTE. All participants were followed up for a duration of up to 48 months. The primary endpoint was cardiovascular mortality, while secondary endpoints comprised the occurrence of ACS, refractory angina pectoris, and cerebrovascular events. Study endpoints were evaluated by dichotomizing the population into two groups using the median CSF value as the cutoff (low: CSF ≤ median; high: CSF > median). **Results:** The primary endpoint did not differ significantly between the two groups; however, refractory angina was significantly more common in the high CSF group [4 (8.2%) vs. 12 (24.5%), *p* = 0.029]. Similarly, the high CSF group exhibited a significantly higher rate of the composite endpoint than the low CSF group [13 (26.5%) vs. 25 (51.0%), *p* = 0.013]. In multivariate Cox analysis, CSF was an independent predictor of the composite endpoint (HR: 1.50; 95% CI: 1.11–2.02, *p* = 0.009). **Conclusions:** Baseline CSF measured by TTE was independently associated with the composite endpoint, whereas its association with isolated ACS was limited. CSF assessment may provide incremental prognostic information alongside conventional arterial and microvascular evaluation.

## 1. Introduction

Acute coronary syndrome (ACS) accounts for the highest rates of mortality and morbidity among cardiovascular diseases, primarily because of its sudden and unpredictable nature [1]. ACS can manifest as unstable angina, non-ST-segment elevation ACS (NSTE-ACS), ST-segment elevation ACS (STE-ACS), and sudden cardiac death [2]. Within the United States, the annual incidence of ACS encompasses approximately 605,000 primary events and 200,000 recurrent episodes; furthermore, an estimated 170,000 cases occur as silent or asymptomatic infarctions [3]. In the United States alone, the annual economic burden of coronary heart disease is estimated to be approximately 46.8 billion dollars [3]. In ACS and, from a broader perspective, coronary artery disease (CAD), the decision-making process remains reliant on the evaluation of coronary anatomy (e.g., stenosis percentage and plaque vulnerability) and arterial physiology (e.g., fractional flow reserve and ischemia testing). The ongoing global impact of ACS outcomes necessitates further advancements in both diagnostic tools and therapeutic approaches [4]. Consequently, research remains focused on discovering new molecules for ACS diagnosis and developing novel therapeutic strategies [5,6,7,8]. The unpredictable nature of ACS has made it a focus of extensive research in recent years, encompassing not only coronary artery pathologies, but also coronary microvascular dysfunction [9]. On the other hand, although interest in the physiological and pathophysiological roles of the coronary sinus (CS) and the coronary venous system has grown, these primary components of cardiac venous drainage in CAD remain relatively under-investigated [10,11]. Recent advancements in non-invasive imaging of the CS have expanded our comprehension of coronary venous anatomy and function, providing a clearer framework for its clinical association with CAD. In recent years, the clinical utility of the CS in treatment strategies has received more attention than its role in disease pathophysiology [12,13]. Coronary circulation functions as an integrated closed-loop system, originating from the epicardial coronary arteries, traversing a vast microvascular network, and culminating in the coronary venous system. The ‘vascular waterfall’ phenomenon is the prevailing hypothesis that explains the dynamics of this closed-loop continuity within the coronary circulatory system [14]. In addition to characterizing physiological flow, the ‘vascular waterfall’ mechanism explains how elevated intramyocardial pressure during systole can lead to a partial cessation of coronary venous return. Given these physiological dynamics, a growing body of literature has focused on the flow metrics of the coronary venous system [15,16]. In particular, CS assessments using transthoracic echocardiography (TTE) are limited and consist of cross-sectional studies.

In light of this information, we aimed to investigate the association between TTE-derived coronary sinus flow (CSF) and the long-term risk of developing ACS.

## 2. Materials and Methods

### 2.1. Study Population

**Figure 1:**
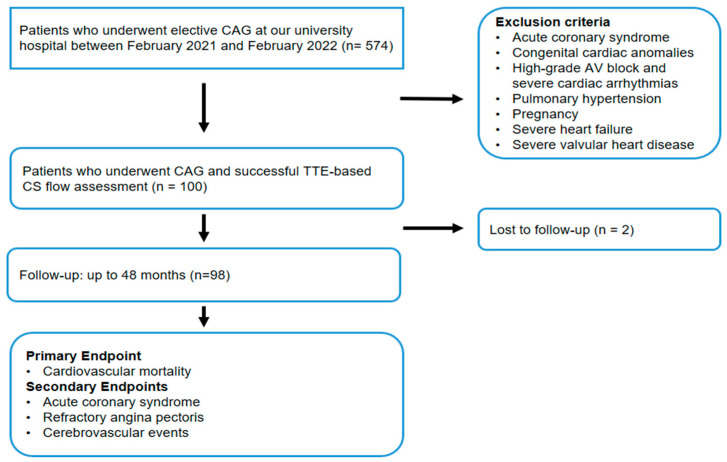
The flowchart of this study. Abbreviations: atrioventricular (AV), coronary angiography (CAG), coronary sinus (CS), transthoracic echocardiography (TTE).

This retrospective cohort study analyzed data from patients previously enrolled in a cross-sectional assessment of CSF using TTE [17]. Patients were eligible for inclusion if they were aged between 18 and 80 years and had undergone coronary angiography (CAG) with a preliminary diagnosis of CAD [18]. Patients were excluded if they met any of the following criteria at the time of initial assessment: pregnancy, ACS, high-grade atrioventricular block and severe cardiac arrhythmias, severe heart failure, congenital cardiac anomalies, pulmonary hypertension, severe valvular heart disease (including prosthetic valves), or suboptimal acoustic windows during TTE examination. Patients with atrial fibrillation were also excluded as CS Doppler parameters were assessed during both systole and diastole, and the absence of organized atrial contraction in these patients precludes accurate measurements [19]. Ultimately, 100 patients who underwent elective diagnostic CAG and successful TTE-based CS flow assessment at our university hospital between February 2021 and February 2022 were enrolled in this study. The study flowchart is illustrated in Figure 1.

### 2.2. Coronary Sinus Flow Measurement

**Figure 2:**
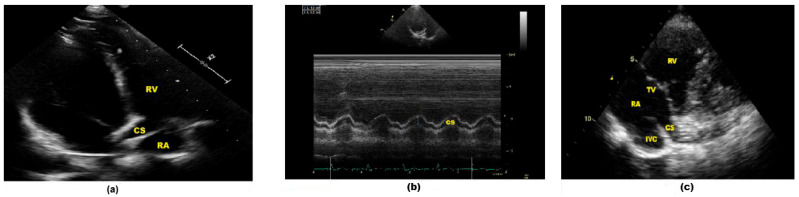
Transthoracic echocardiography-based evaluation of CS flow parameters. (**a**) Modified apical four-chamber imaging allows for clear visualization of the CS opening into the right atrium; (**b**) systolic and diastolic CS diameters were obtained via M-mode in the modified apical four-chamber view; (**c**) entry of the CS into the RA was identified using the modified parasternal long-axis view. Abbreviations: coronary sinus (CS), inferior vena cava (IVC), right atrium (RA), right ventricle (RV), tricuspid valve (TV).

**Figure 3:**
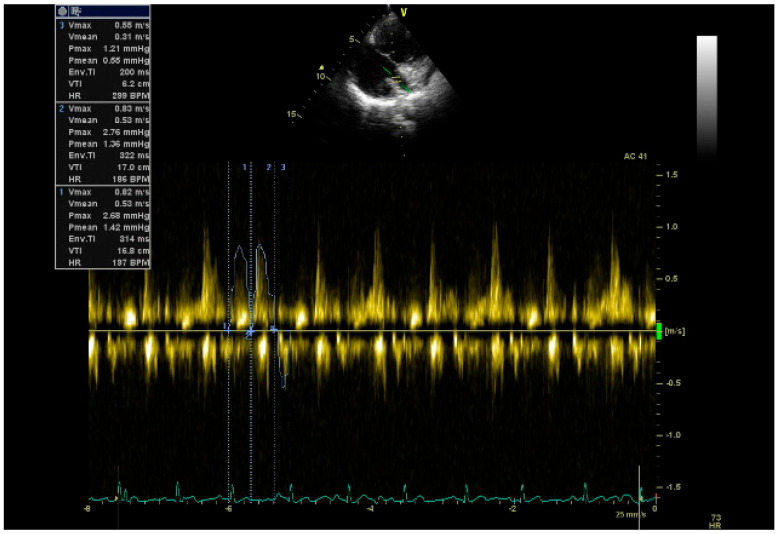
Transthoracic echocardiography-based Doppler measurements of CS flow parameters. In this modified image, PW Doppler measurements reveal a triphasic CS flow pattern: forward systolic and diastolic components, followed by a retrograde diastolic flow representing right atrial contraction. Abbreviations: coronary sinus (CS), pulsed wave (PW).

Comprehensive TTE imaging was carried out with a Philips EPIQ 7 device (Philips Healthcare, Andover, MA, USA), with all echocardiographic parameters calculated according to established international guidelines [20]. Anatomical and functional evaluations of the CS were performed via the parasternal long-axis and modified apical four-chamber views following previously validated protocols [21,22,23]. Specifically, the transducer was tilted posteriorly from this window to clearly visualize the CS as it courses along the posterior atrioventricular groove (Figure 2a). To account for the systolic and diastolic variations in the CS, measurements were performed at the end of diastole, when the vessel reaches its widest point. Additionally, M-mode imaging was utilized within the modified apical four-chamber view to accurately measure both the systolic and diastolic diameters of the CS (Figure 2b). The CS length was determined by averaging three independent measurements to ensure accuracy. In the modified parasternal long-axis view, the CS can be clearly visualized as it drains into the right atrium (Figure 2c). Pulsed-wave (PW) Doppler flow velocities were measured 1 cm from the CS ostium during systole and diastole. In PW Doppler recordings, CS flow is characterized by a triphasic pattern: forward systolic flow, forward diastolic flow, and retrograde diastolic flow, the latter of which reflects right atrial contraction and CS reflux (Figure 3). Coronary sinus flow (CSF) was calculated as the product of the heart rate (HR), CS cross-sectional area, and the sum of the anterograde systolic and diastolic velocity–time integrals (VTIs) following previously established methods in the literature [23]. For the individual standardization of CSF values, the CSF index was obtained by calculating the ratio of the CSF to the body surface area (BSA). Mosteller’s formula was used in the calculation of BSA [24]. Study endpoints were evaluated by dichotomizing the population into two groups based on the median CSF value (low: CSF ≤ median; high: CSF > median).

CS cross-sectional area = *π* × CS diastolic diameter (D)^2^/4

CSF (mL/min) = *π* × D^2^/4 × (VTI_s_ + VTI_d_) × HR

$$BSA(m^{2})=\sqrt{\frac{Height\;\left(cm\right)\times Weight\;\left(kg\right)}{3600}}$$

CSF index (mL/min/m^2^) = CSF (mL/min)/BSA (m^2^)

### 2.3. Assessment of Outcomes

Clinical outcomes were retrospectively evaluated using institutional electronic databases and hospital records. These data were supplemented by telephone interviews with patients or their relatives to document medical history and any clinical symptoms occurring during the follow-up period. The baseline for the follow-up period was defined as the date of the initial TTE and CS flow calculation, from which patients were retrospectively monitored via hospital databases for up to 48 months.

This study is one of the first longitudinal studies in the literature to examine the long-term clinical outcomes of CSF parameters measured by TTE; therefore, cardiovascular mortality was selected a priori as the primary endpoint, given its clinical robustness and objective nature as a hard cardiovascular outcome. We included ACS, cerebrovascular events (CVEs), and refractory angina as secondary outcomes to better reflect the overall clinical burden and provide a more comprehensive assessment of prognostic significance. ACS was defined as the occurrence of acute myocardial infarction (STE or NSTE) and unstable angina pectoris requiring hospitalization. ACS comprised diagnoses of acute myocardial infarction or unstable angina, confirmed by hospital admission records. Myocardial infarction events were defined in accordance with the Fourth Universal Definition of Myocardial Infarction [2]. Refractory angina pectoris was defined as the persistence of symptoms despite optimal, maximally tolerated antianginal therapy, necessitating additional diagnostic evaluation or hospitalization. CVE, including ischemic stroke and transient ischemic attack, were defined based on clinical assessment and diagnostic imaging [1,2,3]. The composite endpoint was pre-specified and defined as a combination of both primary and secondary endpoints. In addition, an exploratory coronary event composite including acute coronary syndrome and refractory angina pectoris was also evaluated in our study. This analysis was conducted to examine the composite burden of coronary ischemic signs in more detail.

The analysis was based on the first clinical outcome recorded for each patient during the follow-up period. Two patients were excluded from the final event analyses due to incomplete medical documentation (electronic health records did confirm that they were alive). Consequently, cardiovascular mortality was assessed in the full cohort of 100 patients, while other clinical outcomes were analyzed in a subset of 98 patients.

### 2.4. Statistical Analysis

The statistical analyses were performed using Jamovi (version 2.3.21, The Jamovi Project). Continuous variables are presented as the mean ± standard deviation (SD) (for normal distributions), while categorical variables are reported as frequencies and percentages [n (%)]. For comparison of continuous variables between two groups, Student’s *t*-test or the Mann–Whitney U test was employed, as appropriate. Categorical variables were compared using the Chi-square test or Fisher’s exact test when expected cell counts were low. Missing data were handled as missing without imputation. The follow-up period was up to 48 months. In our study, we wanted to adopt a pragmatic approach to median-based binary classification given the small sample size of the cohort. The study population was divided into two groups based on the median CSF value (low: CSF ≤ median; high: CSF > median); the event rates are reported for these groups and the *p*-values between them were calculated using the appropriate categorical tests. ROC (receiver operating characteristic) AUC (area under the curve) analysis was performed to evaluate the 48-month discriminatory performance of CS Doppler parameters, and the AUC values are reported. Survival curves for the composite endpoint were generated using the Kaplan–Meier method, and groups defined by the CSF median cutoff were compared using the log-rank test. The graphs displayed censored observations and the number of patients at risk at 0, 12, 24, 36, and 48 months. Cox proportional hazards regression was used for time-dependent outcomes, with results presented as hazard ratios (HRs) and 95% confidence intervals (CIs). For comparability, the effects of CSF and diastolic velocity–time integral (D_VTI) are expressed as the effect per 1 standard deviation (1 SD) increase. We included both CSF and diastolic VTI in the analysis because they reflect coronary venous hemodynamics. CSF is a total parameter encompassing pulse, vessel diameter, and systolic and diastolic flow components, while diastolic VTI physiologically reflects the diastolic component of coronary venous return, which more closely reflects myocardial perfusion. Due to the correlation between CSF and Diastolic VTI, these two variables were not included in the same multivariate model simultaneously to avoid multicollinearity; each was evaluated in separate models. Cox analyses were conducted using complete-case data for relevant outcomes and covariates. In this study, the multivariate model included age, sex, diabetes mellitus, hypertension, current smoking status, and hyperlipidemia, as these variables are clinically relevant predictors of cardiovascular risk and potential confounders. All tests were two-sided, and a *p*-value < 0.05 was considered statistically significant.

## 3. Results

In this retrospective observational cohort study, the initial cohort comprised 100 patients. The mean age of the study participants was 59.08 ± 11.57 years, and 39% were female. In the study population, the mean CSF value was 374.74 ± 171.76 mL/min, while the CSF index was 198.50 ± 95.83 mL/min/m^2^. Low and high CSF groups were defined using the median CSF value as the cutoff (355.69 mL/min). Baseline sociodemographics, laboratory and medical therapy by CSF group are summarized in Table 1.

Eight mortalities occurred during follow-up: seven were due to cardiovascular mortality associated with ACS (type 3 myocardial infarction) [2] and one resulted from cancer. The primary endpoint did not differ significantly between the two groups; however, the secondary endpoint of refractory angina was significantly more common in the high CSF group [4 (8.2%) vs. 12 (24.5%), *p* = 0.029]. Similarly, the high CSF group exhibited a significantly higher rate of the composite endpoint than the low CSF group [13 (26.5%) vs. 25 (51.0%), *p* = 0.013] (Table 2).

The relationship between CS Doppler parameters and clinical endpoints over the 48-month follow-up was analyzed using ROC-AUC analysis and the results are presented in Table 3. The best AUC values are shown in bold for each endpoint. Refractory angina and ACS—representing all coronary events—are presented in a separate column in Table 3. The analysis revealed that the CSF parameter yielded the highest overall predictive value, particularly for forecasting refractory angina and the development of ACS (AUC = 0.679), as well as for the composite endpoint (AUC = 0.656). Regarding predicting cardiovascular mortality, diastolic velocity parameters (D_Vmean and D_VTI) showed a modest discriminatory ability (AUC = 0.635 and 0.628). In contrast, the association of systolic parameters (S_Vmax and S_Vmean) with clinical endpoints was more limited compared to diastolic parameters. None of the parameters reached the AUC threshold of 0.70, indicating that while CS Doppler parameters provide some prognostic information, their individual power to predict clinical outcomes remains modest.

In this cohort, multivariable Cox proportional hazards analysis found that CSF was not independently associated with cardiovascular mortality (HR = 0.87, 95% CI: 0.35–2.20, *p* = 0.774) or ACS development (HR = 1.13, 95% CI: 0.68–1.88, *p* = 0.638). On the other hand, CSF was found to be a significant independent predictor of the composite endpoint (HR = 1.50, 95% CI: 1.11–2.02, *p* = 0.009). Furthermore, age was identified as the only other significant covariate for cardiovascular mortality (HR = 1.10, 95% CI: 1.01–1.19, *p* = 0.021) (Table 4). In the multivariate Cox models, diastolic VTI was not independently associated with cardiovascular mortality (HR = 1.66; 95% CI: 0.69–4.00; *p* = 0.260) or ACS (HR = 1.03; 95% CI: 0.60–1.77; *p* = 0.915). Age emerged as the sole significant predictor of cardiovascular mortality (HR = 1.09; 95% CI: 1.00–1.19; *p* = 0.043). Regarding the composite endpoint, diastolic VTI exhibited a borderline non-significant trend toward an increased risk (HR = 1.43; 95% CI: 0.98–2.08; *p* = 0.061), suggesting that its prognostic contribution remains limited in our study population (Table 5).

**Figure 4:**
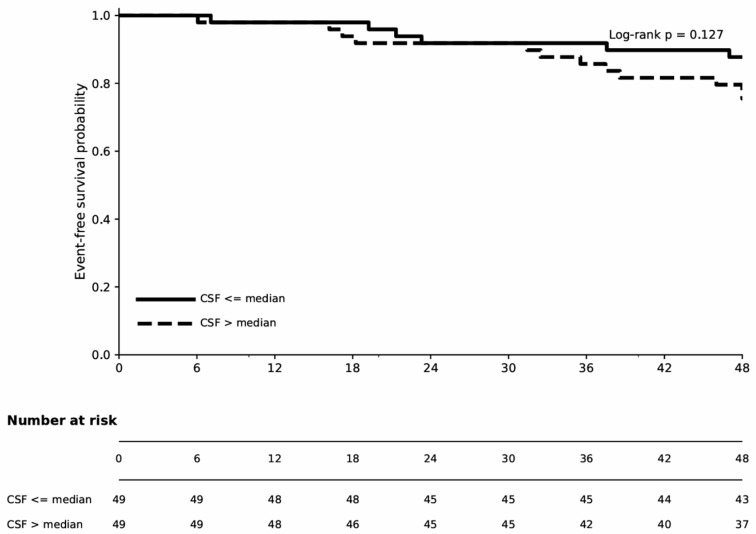
Kaplan–Meier curves for acute coronary syndrome in CSF groups (≤median vs. >median). The *p*-value was calculated using the log-rank test (*p* = 0.127). Abbreviations: coronary sinus flow (CSF).

**Figure 5:**
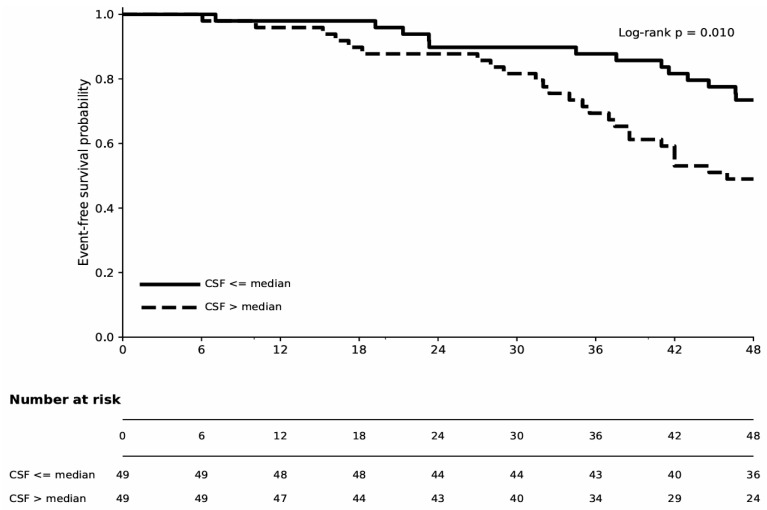
Kaplan–Meier curves for the composite endpoint in CSF groups (≤median vs. >median). Number at risk and log-rank *p*-value are shown in the figure (*p* = 0.01). Abbreviations: coronary sinus flow (CSF).

Kaplan–Meier analysis demonstrated that the high CSF group had a higher rate of ACS compared to the low CSF group; however, this difference did not reach statistical significance (log-rank *p* = 0.127). These findings suggest that CSF alone may have limited utility in stratifying ACS risk over a 48-month period (Figure 4). Kaplan–Meier analysis of the composite endpoint revealed a significantly higher composite event rate in the high CSF group (log-rank *p* = 0.01). Consistent with the multivariate Cox analysis, this finding supports the independent prognostic value of CSF for predicting the composite outcome (Figure 5).

## 4. Discussion

Our study demonstrates that, although baseline CSF can be a predictor of composite cardiovascular endpoints in stable CAD, it shows limited prognostic utility for ACS alone. This study has a small sample size and the limited number of ACS events may have resulted in insufficient statistical power for differences in CSF parameters to reach significance; however, the higher event rate for the composite endpoint likely enabled the detection of a statistically significant association. Kanaji et al. established that cardiac magnetic resonance (CMR)-derived CSF is a significant prognostic indicator post-acute myocardial infarction, independent of conventional risk factors and infarct size [25]. In that study, CMR imaging was performed approximately 30 days post-myocardial infarction. Although their findings align with ours, CMR remains a relatively time-consuming and costly procedure, with potential contraindications that may limit its clinical utility in certain patient populations. Recent meta-analytic evidence involving cardiac MRI indicates that patients with cardiovascular disease exhibit markedly lower CSF levels than healthy individuals [26]. Unlike previous CMR-based meta-analyses, our finding of a link between higher CSF and adverse outcomes likely reflects the specific composition of our study population, which lacked a healthy control group, and may be further influenced by the limited number of events. To the best of our knowledge, this is the first study to evaluate TTE-derived CSF in relation to long-term ACS risk. While previous research was primarily cross-sectional or physiological in nature, our study extends these findings by providing longitudinal clinical follow-up data. Lyubarova et al. evaluated TTE-derived CSF levels in 24 patients and reported significantly higher values among those undergoing percutaneous coronary intervention (PCI) [23]. As diagnostic tools have improved, microvascular disease has not only become better understood, but research on the coronary venous system has also intensified. A recently published article correlated CSF measurements, performed using dynamic coronary computed tomography (CT) angiography—a novel non-invasive method—with myocardial flow reserve measurements obtained from PET [27]. Although CSF measurement in cardiac CT angiography imaging is a new method, these imaging methods cannot be used in clinical practice to evaluate CS flow parameters because CT exposes patients to significant amounts of radiation. From this perspective, TTE is an easily repeatable, inexpensive, and radiation-free imaging method for evaluating CSF.

Refractory angina pectoris is a challenging clinical condition in which anginal symptoms persist despite optimal medical treatment, and there are few further revascularization options [28]. Given the clinical implementation of CS reducer devices, the relationship between CS flow dynamics and refractory angina becomes even more critical for optimizing patient selection and treatment outcomes [28,29]. The observed relationship between coronary venous flow parameters and refractory angina can provide valuable insights for understanding and evaluating the efficacy of novel therapeutic strategies targeting the coronary venous system [30,31]. In addition to the therapeutic intervention using the coronary venous system, there have also been discussions on whether changes in CS Doppler parameters have clinical consequences [32]. Notwithstanding progress in medical and procedural interventions, approximately 40% of patients with chronic stable angina continue to experience persistent symptoms despite receiving optimal medical treatment and undergoing revascularization [30]. Furthermore, considering that approximately 30% of patients treated with CS reducer devices are non-responders [31], characterizing baseline CS flow profiles prior to implantation may help identify suitable candidates and potentially reduce non-response rates. The higher CSF values observed in our study, particularly in patients with refractory angina, necessitate broader clinical and experimental research to determine whether this reflects increased resistance in the microvascular bed or is a consequence of coronary venous congestion. The findings of our study can also be interpreted in terms of the multifaceted pathophysiology of ACS. The onset and severity of ACS can be influenced not only by major coronary arteries, but also by microvascular disease, critical myocardial supply–demand imbalances, and neurohumoral mechanisms. Previous studies have shown that markers of metabolic pathway activity can provide additional prognostic clinical information in ACS. For example, serum gamma-glutamyltransferase (GGT) and uric acid levels are known to be important for prognostic assessment in ACS; notably, one study showed that in NSTEMI patients, early initiation of metoprolol and carvedilol did not change serum GGT and uric acid levels [33]. This study supports the view that adverse cardiovascular outcomes may result from a complex interplay between ischemic, hemodynamic, and metabolic events. In this context, CSF can be considered another complementary physiological indicator within this broad cardiovascular framework.

The circulation of the heart, starting from the major arteries, continuing through the coronary microvascular bed, and finally ending in the coronary veins, is explained by the vascular waterfall mechanism. However, it has been experimentally shown that coronary perfusion is impaired through this same mechanism after a certain systolic pressure occurs [14]. Although the microvascular bed blood supply improves with increasing coronary venous pressure up to a certain point, at higher pressures, the increased resistance in the microvascular bed can impair coronary arterial flow. This can lead to a long-term imbalance in coronary supply and demand, contributing to refractory angina and poor cardiovascular outcomes in cases of poor remodeling. The elevated CSF measured at baseline may be more of a compensatory response to microvascular dysfunction or increased myocardial oxygen demand than a direct causal mechanism in and of itself. Furthermore, without further support from invasive physiological and intravascular imaging methods, particularly those examining the microvascular bed, this observed relationship should be interpreted as hypothesis-generating rather than a definitive hemodynamic relationship. Well-designed prospective clinical, hemodynamic, and experimental studies are needed to validate these theories.

The primary limitation of this study is that only baseline CS flow parameters were available, and we were unable to assess longitudinal changes in CS flow during the follow-up period. Furthermore, the dynamics of the coronary microcirculation, which constitutes an extensive network within the coronary system, were not directly assessed in our study. Utilizing diagnostic modalities such as coronary flow reserve to rule out concomitant microvascular disease could allow for a clearer isolation of the specific impact of coronary venous hemodynamics on CAD. Additionally, as patients were free of ACS at the time of enrollment, only baseline CSF parameters were compared with subsequent ACS events; furthermore, sub-analyses were not performed for patients undergoing revascularization during follow-up, such as PCI or coronary artery bypass grafting. Another significant limitation is the retrospective design and the relatively small sample size given the number of covariates included in the multivariate Cox proportional hazards models. Although major cardiovascular risk factors were adjusted in order to isolate the independent prognostic value of CSF, the low events-per-variable ratio may increase the risk of overfitting. Consequently, while our findings provide compelling preliminary evidence for the use of CSF to predict the composite endpoint, the associated hazard ratio estimates may exhibit instability. To avoid multicollinearity and overfitting in a small cohort, CSF and diastolic VTI were analyzed in separate multivariate models rather than combined. Additionally, the study design did not include a formal power calculation for predicting clinical outcomes. Specifically, investigating ‘cardiovascular mortality’ as a primary endpoint in larger cohorts will clarify our understanding of this issue. Therefore, these results should be considered exploratory and hypothesis-generating rather than confirmatory. Larger, prospective multicenter studies are warranted to validate these findings and allow for more robust modeling with a higher number of clinical events. In addition, future studies should analyze CSF as a continuous variable and investigate threshold values derived from ROCs.

## 5. Conclusions

The findings of this study suggest that baseline CSF measured by TTE is independently associated with the composite endpoint, whereas its association with isolated ACS is limited. CSF assessment may provide incremental prognostic information alongside conventional arterial and microvascular evaluation.

## Figures and Tables

**Table 1:** Baseline characteristics, laboratory and medical therapy by coronary sinus flow group.

Variable	All (n = 100)	Low CSF (≤Median) (n = 50)	High CSF (>Median) (n = 50)	*p*-Value
**Demographics, clinical status, and laboratory values**				
Age, years	59.08 ± 11.57	57.73 ± 12.89	60.41 ± 10.71	0.262
BSA, m^2^	1.93 ± 0.37	1.92 ± 0.45	1.95 ± 0.36	0.715
LVEF, %	58.38 ± 3.64	59.09 ± 3.73	57.98 ± 3.60	0.132
Hemoglobin, g/dL	13.30 ± 1.86	13.44 ± 1.70	12.98 ± 2.01	0.214
eGFR, mL/min/1.73 m^2^	83.96 ± 19.67	84.75 ± 17.34	79.46 ± 23.85	0.209
**Cardiometabolic risk factors**				
Gender, female, n (%)	39 (39.0)	18 (36.0)	21 (42.0)	0.670
Diabetes mellitus, n (%)	33 (33.0)	16 (32.0)	17 (34.0)	1.000
Hypertension, n (%)	43 (43.0)	23 (46.0)	20 (40.0)	0.682
Current smoking, n (%)	55 (55.0)	28 (56.0)	27 (54.0)	1.000
Hyperlipidemia, n (%)	40 (40.0)	21 (42.0)	19 (38.0)	0.840
**Medications**				
Antiplatelet, n (%)	62 (62.0)	28 (56.0)	34 (68.0)	0.340
Statin, n (%)	58 (58.0)	33 (66.0)	25 (50.0)	0.160
Beta-blocker, n (%)	48 (48.0)	26 (52.0)	22 (44.0)	0.543
ACEi/ARB, n (%)	58 (58.0)	30 (60.0)	28 (56.0)	0.850
Nitrate, n (%)	4 (4.0)	2 (4.0)	2 (4.0)	1.000
Ranolazine, n (%)	8 (8.0)	5 (10.0)	3 (6.0)	0.715
Trimetazidine, n (%)	5 (5.0)	3 (6.0)	2 (4.0)	1.000
SGLT2 inhibitor, n (%)	8 (8.0)	3 (6.0)	5 (10.0)	0.715

Low and high CSF groups were defined using the median CSF value as the cutoff (355.69 mL/min). Data are presented as mean ± SD for continuous variables. Missing values were kept as missing (no imputation). Percentages for medications were calculated based on available follow-up medication data; denominators may vary. Abbreviations: angiotensin-converting enzyme inhibitor (ACEi), angiotensin receptor blocker (ARB), body surface area (BSA), coronary sinus flow (CSF), estimated glomerular filtration rate (eGFR), left ventricular ejection fraction (LVEF), sodium–glucose cotransporter-2 (SGLT2), standard deviation (SD).

**Table 2:** Event rates in CSF groups.

Endpoint	Low CSF (≤Median)	High CSF (>Median)	*p*
**Primary endpoint**			
Cardiovascular mortality	3 (6.0%)	4 (8.0%)	0.695
**Secondary endpoints**			
Acute coronary syndrome	6 (12.2%)	12 (24.5%)	0.118
Cerebrovascular event	3 (6.1%)	1 (2.0%)	0.617
Refractory angina	4 (8.2%)	12 (24.5%)	0.029 *
**Composite endpoint**	13 (26.5%)	25 (51.0%)	0.013 *

Values are n (%). Low and high CSF groups were defined using the median CSF value as the cutoff (355.69 mL/min). Percentages were calculated using complete-case denominators for each endpoint (mortality: n = 50 per group; composite endpoint: n = 49 per group). Abbreviations: coronary sinus flow (CSF). * *p* < 0.05.

**Table 3:** Predictive values of coronary sinus Doppler parameters for 48-month clinical outcomes (ROC-AUC analysis).

Parameter	Cardiovascular Mortality	ACS and Refractory Angina	Composite Endpoint
Diastolic Vmax	0.508	0.538	0.518
Diastolic Vmean	**0.635**	0.561	0.583
Diastolic VTI	0.628	0.609	0.630
Systolic Vmax	0.524	0.531	0.531
Systolic Vmean	0.533	0.554	0.554
Systolic VTI	0.605	0.625	0.583
Coronary Sinus Flow	0.470	**0.679**	**0.656**

Best AUC values are shown in **bold** for each endpoint. Abbreviations: acute coronary syndrome (ACS), area under the curve (AUC), mean velocity (Vmean), peak velocity (Vmax), receiver operating characteristic (ROC), velocity–time integral (VTI).

**Table 4:** Multivariable Cox proportional hazards models including coronary sinus flow.

Outcome	Variable	HR (95% CI)	*p*-Value
Cardiovascular mortality (n = 100; events = 7)			
	CSF (per 1 SD)	0.87 (0.35–2.20)	0.774
	Age (per 1 year)	1.10 (1.01–1.19)	0.021 *
	Male sex (vs. female)	0.76 (0.14–4.19)	0.757
	Diabetes mellitus	3.25 (0.38–27.52)	0.280
	Hypertension	0.52 (0.11–2.50)	0.413
	Current smoker	0.29 (0.05–1.73)	0.175
	Hyperlipidemia	1.47 (0.27–7.97)	0.656
ACS (n = 98; events = 18)			
	CSF (per 1 SD)	1.13 (0.68–1.88)	0.638
	Age (per 1 year)	1.03 (0.98–1.08)	0.235
	Male sex (vs. female)	0.92 (0.34–2.44)	0.861
	Diabetes mellitus	2.06 (0.66–6.46)	0.215
	Hypertension	0.64 (0.25–1.63)	0.352
	Current smoker	1.31 (0.47–3.68)	0.605
	Hyperlipidemia	1.40 (0.51–3.86)	0.516
Composite endpoint (n = 98; events = 38)			
	CSF (per 1 SD)	1.50 (1.11–2.02)	0.009 *
	Age (per 1 year)	1.03 (1.00–1.07)	0.086
	Male sex (vs. female)	0.93 (0.47–1.82)	0.828
	Diabetes mellitus	0.60 (0.31–1.16)	0.129
	Hypertension	1.04 (0.53–2.01)	0.915
	Current smoker	1.07 (0.50–2.25)	0.868
	Hyperlipidemia	0.93 (0.48–1.81)	0.827

Models include CSF (per 1 SD increase), age, sex, diabetes mellitus, hypertension, current smoker, and hyperlipidemia. HRs represent relative hazards per 1 SD increase in CSF. CSF and diastolic VTI were evaluated in separate models due to their correlation (Table 4 and Table 5). Two-sided *p*-values are shown; ties were handled using Breslow’s method; complete-case analysis was performed for each endpoint. Abbreviations: acute coronary syndrome (ACS); confidence interval (CI), coronary sinus flow (CSF), hazard ratio (HR), standard deviation (SD). * *p* < 0.05.

**Table 5:** Multivariable Cox proportional hazards models including diastolic VTI.

Outcome	Variable	HR (95% CI)	*p*-Value
Cardiovascular mortality (n = 100; events = 7)			
	Diastolic VTI (per 1 SD)	1.66 (0.69–4.00)	0.260
	Age (per 1 year)	1.09 (1.00–1.19)	0.043 *
	Male sex (vs. female)	0.45 (0.06–3.32)	0.435
	Diabetes mellitus	3.97 (0.45–34.68)	0.213
	Hypertension	0.71 (0.13–3.84)	0.690
	Current smoker	0.23 (0.04–1.43)	0.116
	Hyperlipidemia	1.62 (0.30–8.90)	0.578
ACS (n = 98; events = 18)			
	Diastolic VTI (per 1 SD)	1.03 (0.60–1.77)	0.915
	Age (per 1 year)	1.03 (0.98–1.09)	0.276
	Male sex (vs. female)	0.90 (0.31–2.63)	0.847
	Diabetes mellitus	2.02 (0.65–6.31)	0.225
	Hypertension	0.65 (0.25–1.69)	0.377
	Current smoker	1.35 (0.48–3.75)	0.567
	Hyperlipidemia	1.35 (0.49–3.70)	0.561
Composite endpoint (n = 98; events = 38)			
	Diastolic VTI (per 1 SD)	1.43 (0.98–2.08)	0.061
	Age (per 1 year)	1.03 (0.99–1.07)	0.209
	Male sex (vs. female)	0.75 (0.36–1.57)	0.445
	Diabetes mellitus	0.58 (0.30–1.12)	0.104
	Hypertension	1.19 (0.61–2.33)	0.608
	Current smoker	1.23 (0.59–2.54)	0.584
	Hyperlipidemia	0.89 (0.45–1.73)	0.721

Models include diastolic VTI (per 1 SD increase), age, sex, diabetes mellitus, hypertension, current smoker, and hyperlipidemia. HRs represent relative hazards per 1 SD increase in diastolic VTI. CSF and diastolic VTI were evaluated in separate models due to their correlation (Table 4 and Table 5). Two-sided *p*-values are shown; ties were handled using Breslow’s method; complete-case analysis was performed for each endpoint. Abbreviations: ACS, acute coronary syndrome; CI, confidence interval; HR, hazard ratio; SD, standard deviation; VTI, velocity–time integral. * *p* < 0.05.

## Data Availability

The data used in this study will be shared upon reasonable request to the corresponding author.

## Abbreviations

The following abbreviations are used in this manuscript:

ACS	Acute coronary syndrome
AUC	Area under the curve
BSA	Body surface area
CMR	Cardiac magnetic resonance
CVE	Cerebrovascular event
CT	Computed tomography
CI	Confidence interval
CAG	Coronary angiography
CAD	Coronary artery disease
CS	Coronary sinus
CSF	Coronary sinus flow
HR	Hazard ratio
NSTE	Non-ST-segment elevation
PCI	Percutaneous coronary intervention
PW	Pulsed wave
ROC	Receiver operating characteristic
STE	ST-segment elevation
TTE	Transthoracic echocardiography
VTI	Velocity–time integral
